# Supplementary material for: Contrasting life-history responses to climate variability in eastern and western North Pacific sardine populations
Source: Nat Commun. 2022 Oct 16;13:5298. doi: 10.1038/s41467-022-33019-z (PMC9573866; doi:10.1038/s41467-022-33019-z)
Supplement: Supplementary file 1 — Supplementary Information [file 41467_2022_33019_MOESM1_ESM.pdf]

Supplementary Information for

Contrasting life-history responses to climate variability in eastern and western North Pacific sardine populations

Authors

Tatsuya Sakamoto[1*], Motomitsu Takahashi[1], Ming-Tsung Chung[2,3], Ryan R. Rykaczewski[4,5], Kosei Komatsu[6,2], Kotaro Shirai[2], Toyoho Ishimura[7,8], Tomihiko Higuchi[2]

Affiliations

1. Japan Fisheries Research and Education Agency, Nagasaki, Japan
2. Atmosphere and Ocean Research Institute, The University of Tokyo, Chiba, Japan
3. Institute of Oceanography, National Taiwan University, Taipei, Taiwan
4. Pacific Islands Fisheries Science Center, NOAA National Marine Fisheries Service, Honolulu, HI, USA
5. Department of Oceanography, School of Ocean and Earth Science and Technology, University of Hawaii, Honolulu, HI, USA
6. Graduate School of Frontier Sciences, University of Tokyo, Chiba, Japan
7. Graduate School of Human and Environmental Studies, Kyoto University, Kyoto, Japan
8. Department of Chemistry and Material Engineering, National Institute of Technology, Ibaraki College, Ibaraki, Japan

*Corresponding author

Email: tatsfish@gmail.com

Present address: Instituto Português do Mar e da Atmosfera (IPMA), Rua Alfredo Magalhães Ramalho, 6, 1495-006 Lisbon, Portugal

Supplementary Information:

Supplementary Figure 1–12

Supplementary Table 1–10

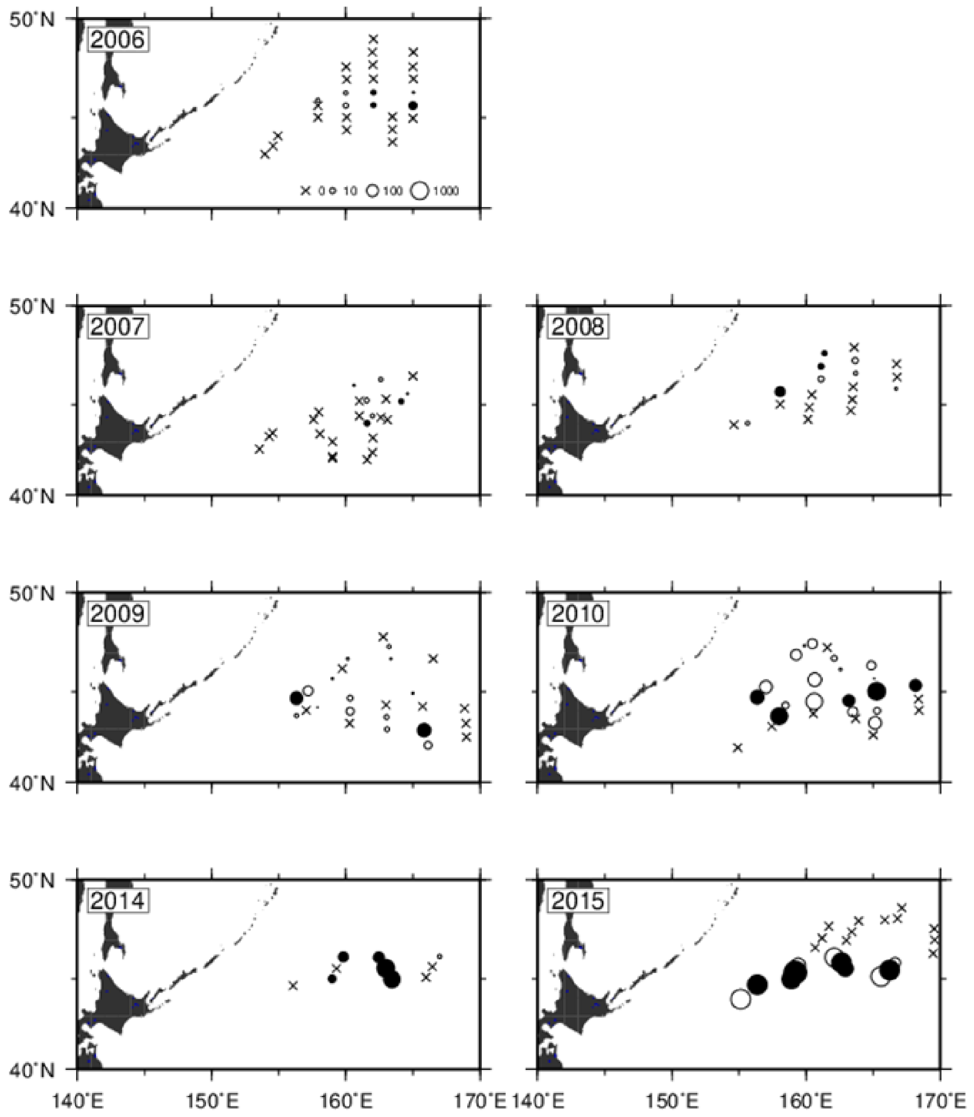

Supplementary Figure 1. Sampling locations of JP sardine. Results of trawl surveys for JP sardine in the offshore Oyashio region during 2006–2010 and 2014–2015. The size of each circle represents CPUE (the number of age-0 sardine captured per 30-minute tow), and filled circles are the stations from which we collected otoliths for this study.

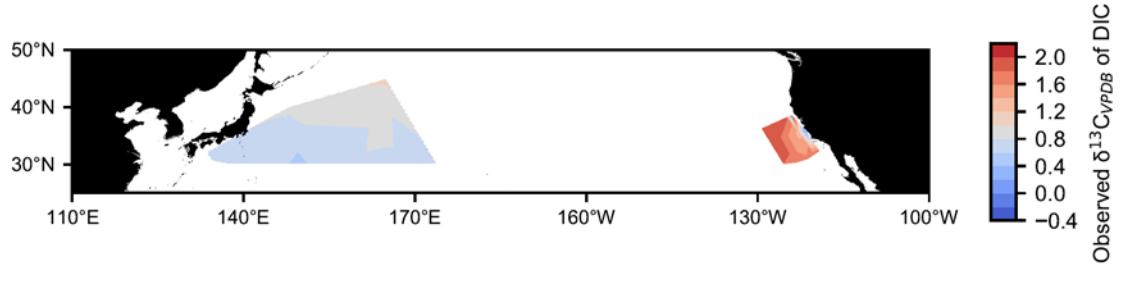

Supplementary Figure 2. Horizontal distribution of δ^{13}C of DIC in surface layer (> 50m) seawater. Measurements from the Kuroshio-Oyashio system during 2006–2015 and from the California Current system during 1986–2006 are used.

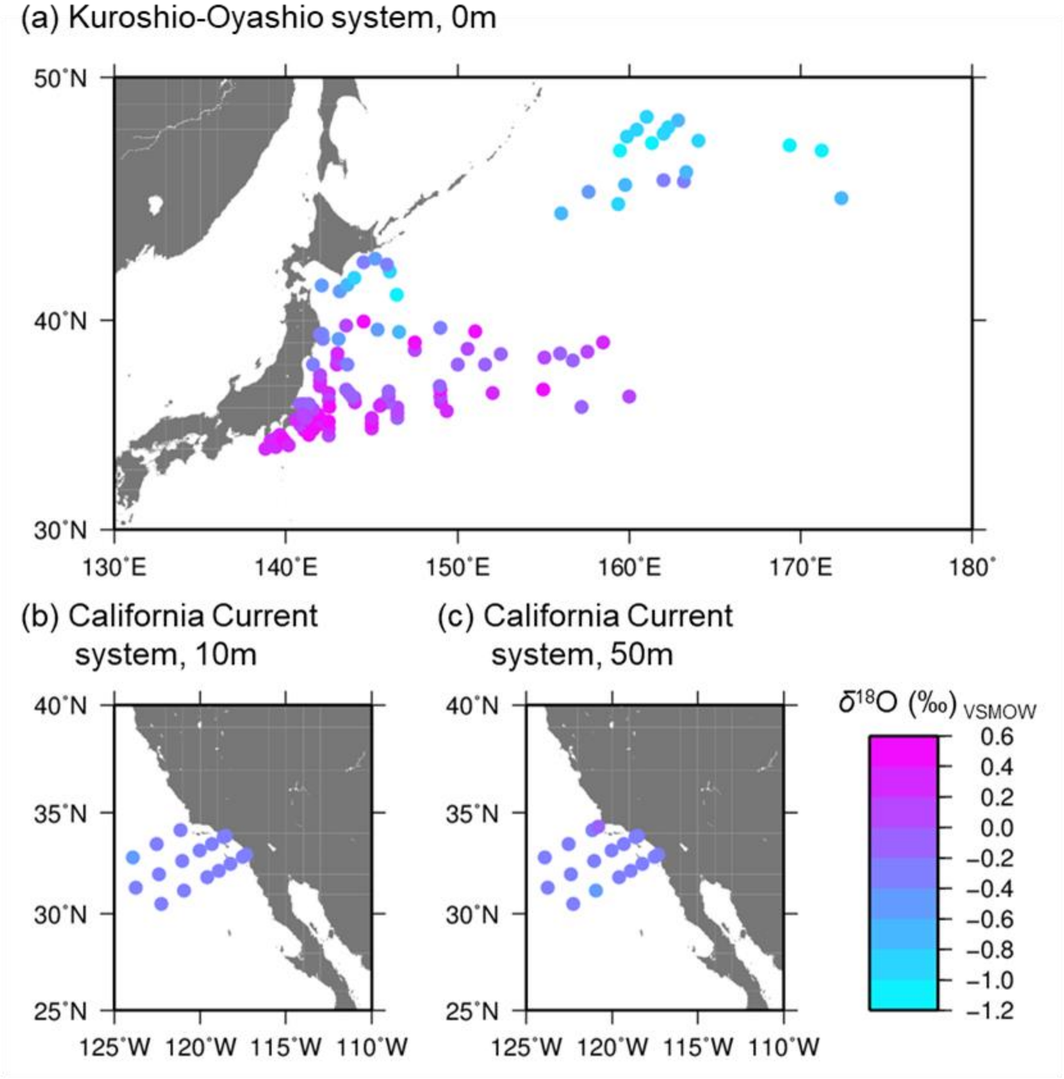

Supplementary Figure 3. Horizontal distribution of seawater δ^{18}O. Seawater δ^{18}O in (a) the Kuroshio-Oyashio system at surface (≤ 5m) redrawn from Sakamoto et al.[39] and (b) the California Current system at 10m and (c) 50m are shown. Note that colour scales are common.

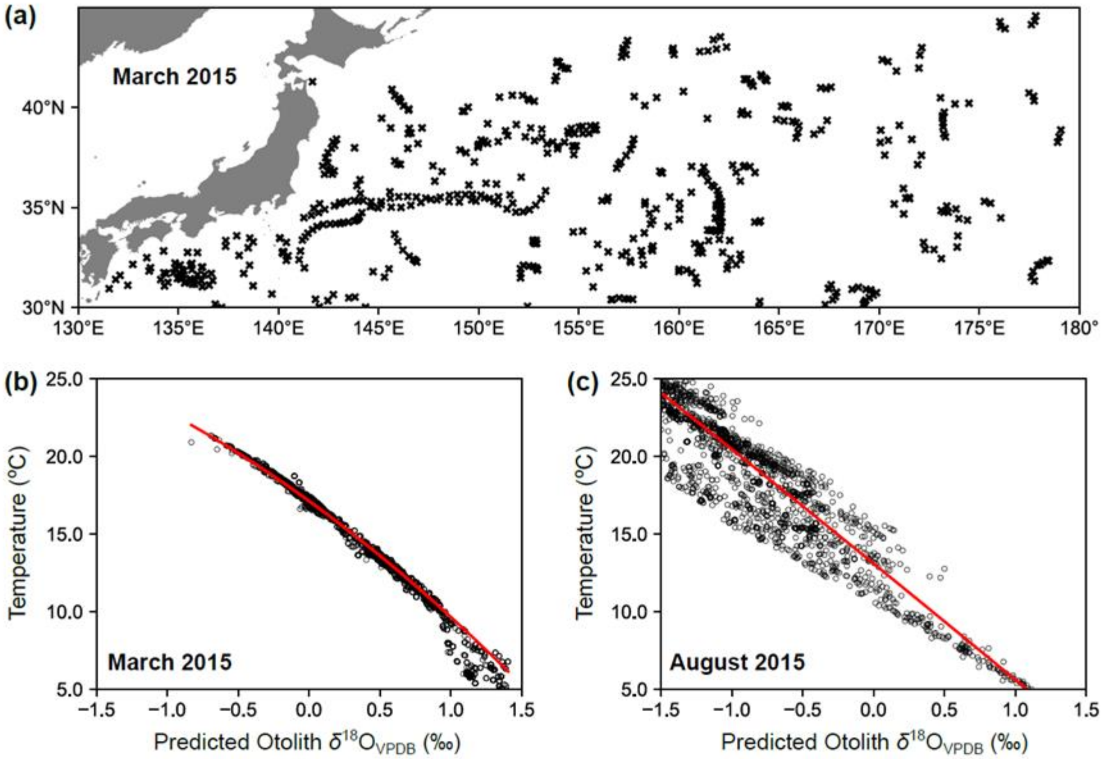

Supplementary Figure 4. Examples of Argo float data used for conversion of otolith δ^{18}O to temperature. (a) Observation locations of Argo floats in March 2015 and (b) the relationship between temperature and otolith δ^{18}O predicted from temperature and salinity observations during the month. The quadratic regression line is shown in red. (c) The relationship between temperature and predicted otolith δ^{18}O for August 2015, which shows larger variations from the regression line.

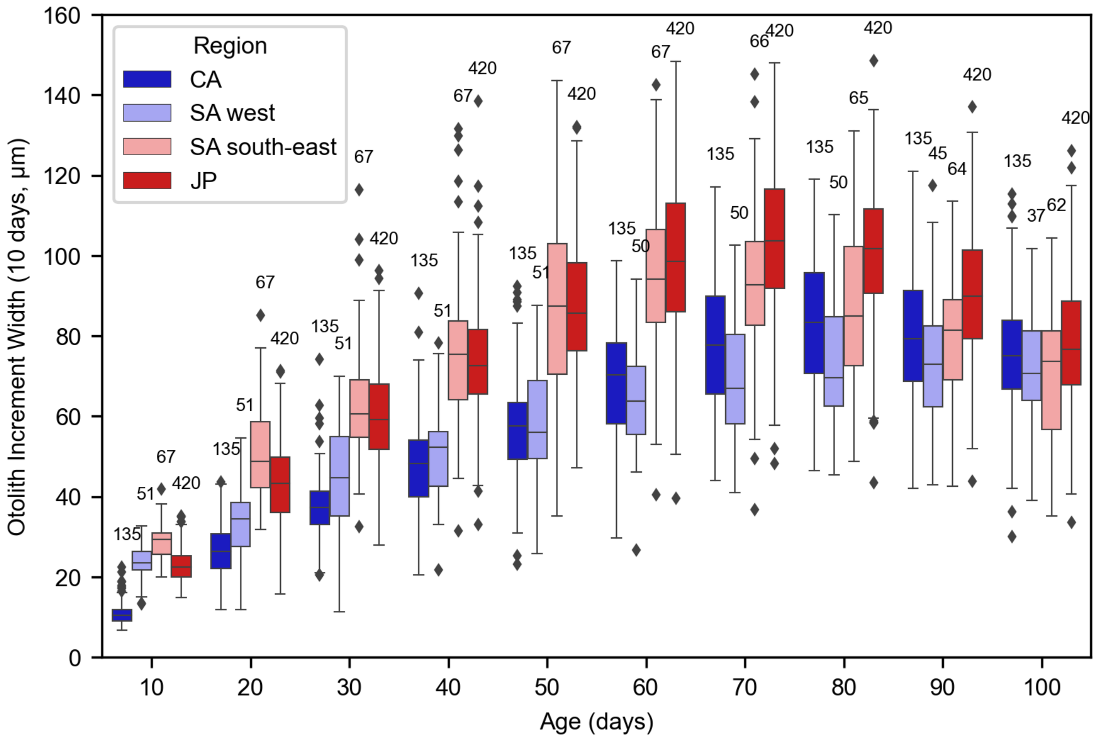

Supplementary Figure 5. **Ten days otolith increment widths of JP, CA and South African sardines.** The values at age 10**n* (n > 1) show the total otolith increment during 10**n*-9 to 10**n* dph, and values at age 10 show the otolith radius at 10 dph. Center line, median; box limits, upper and lower quartiles; whiskers, 1.5x interquartile range; points, outliers. The numbers show the number of individuals for each boxplot.

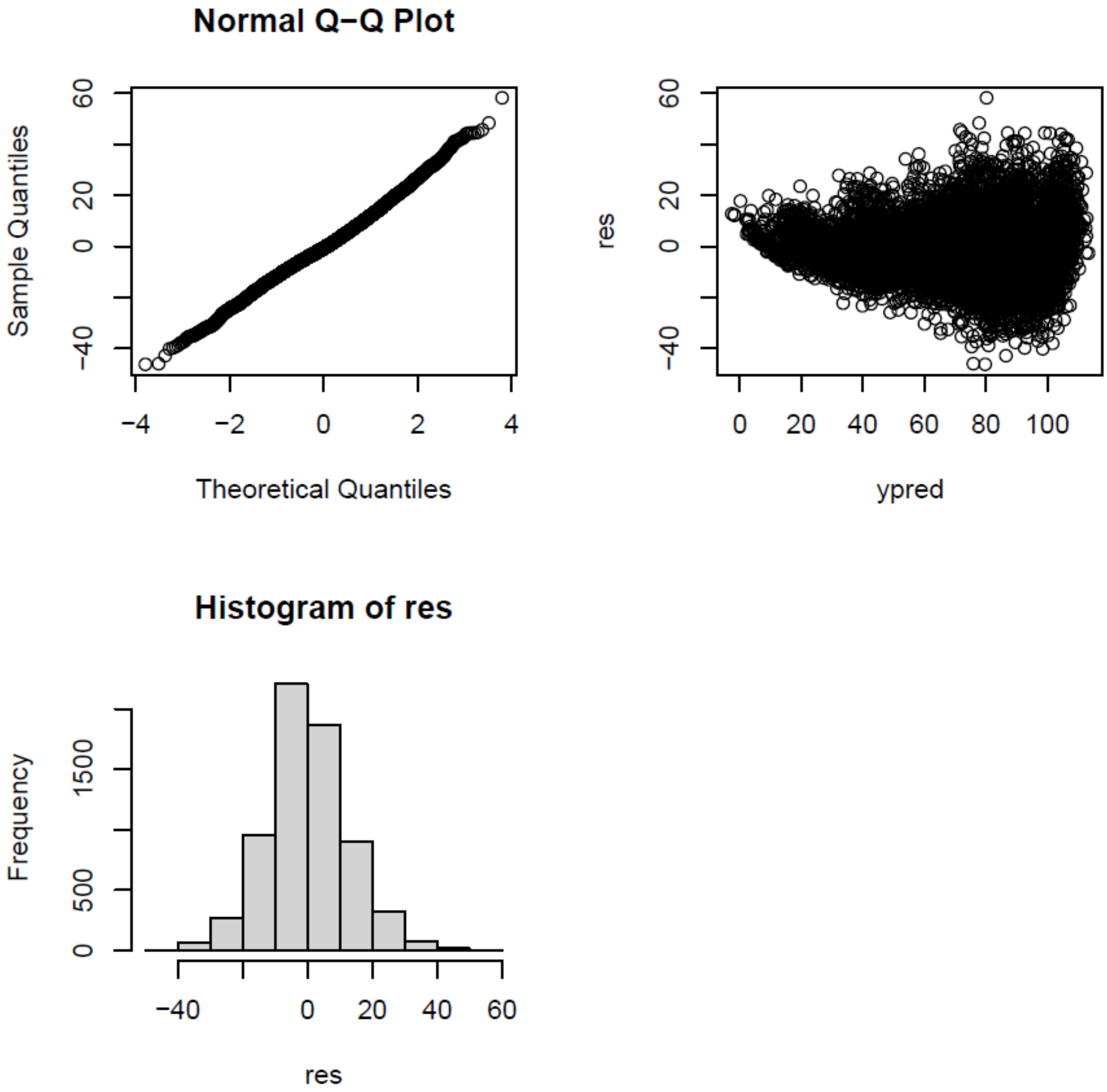

Supplementary Figure 6. **The diagnostic of the linear mixed-effects model (lmer(*IW ~ Age*Region* + (1 | *Fish.ID*))).**

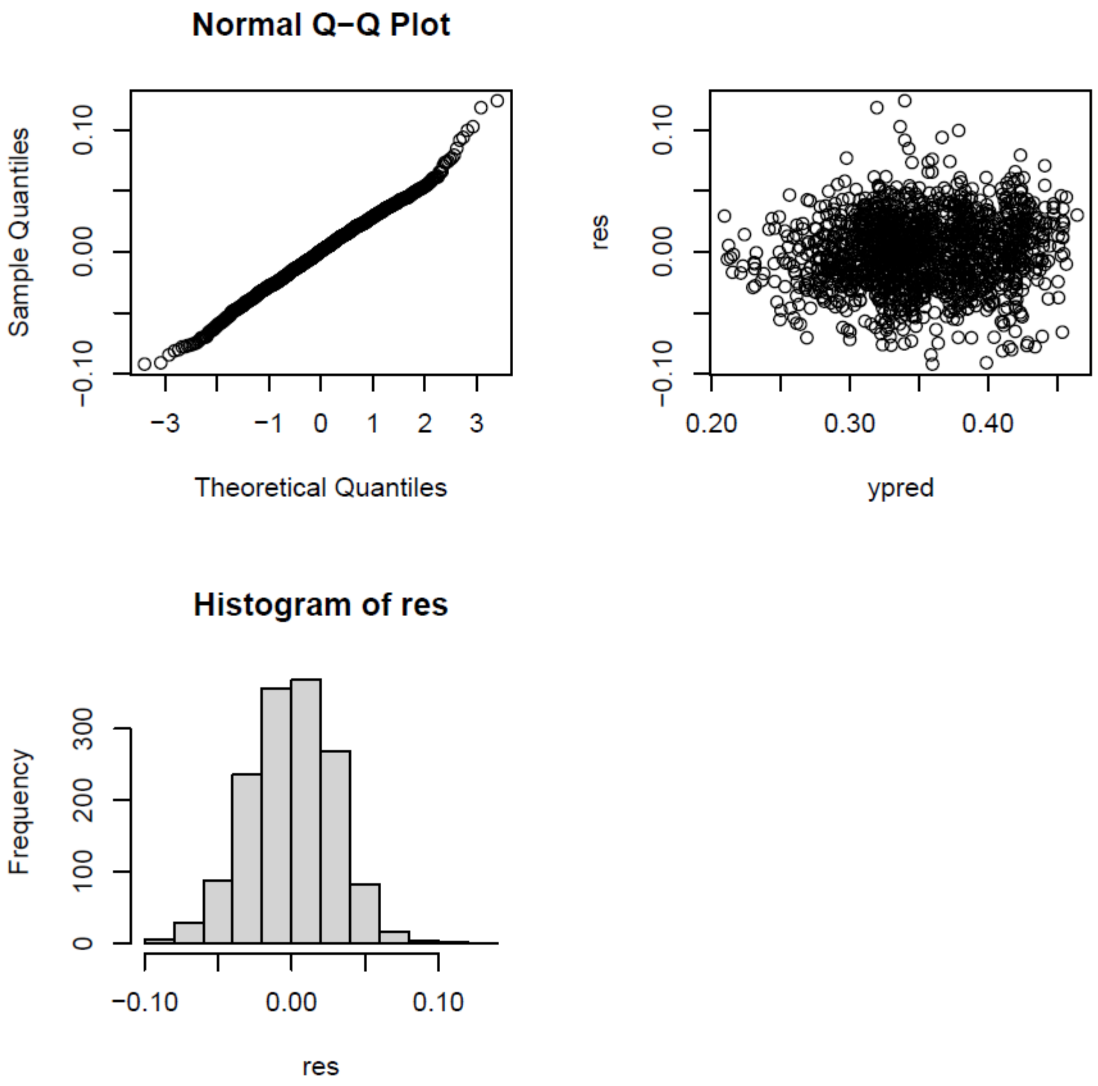

Supplementary Figure 7. The diagnostic of the linear mixed-effects model (lmer(M_{oto} ~ *Age*Region* + (1 | *Fish.ID*))).**

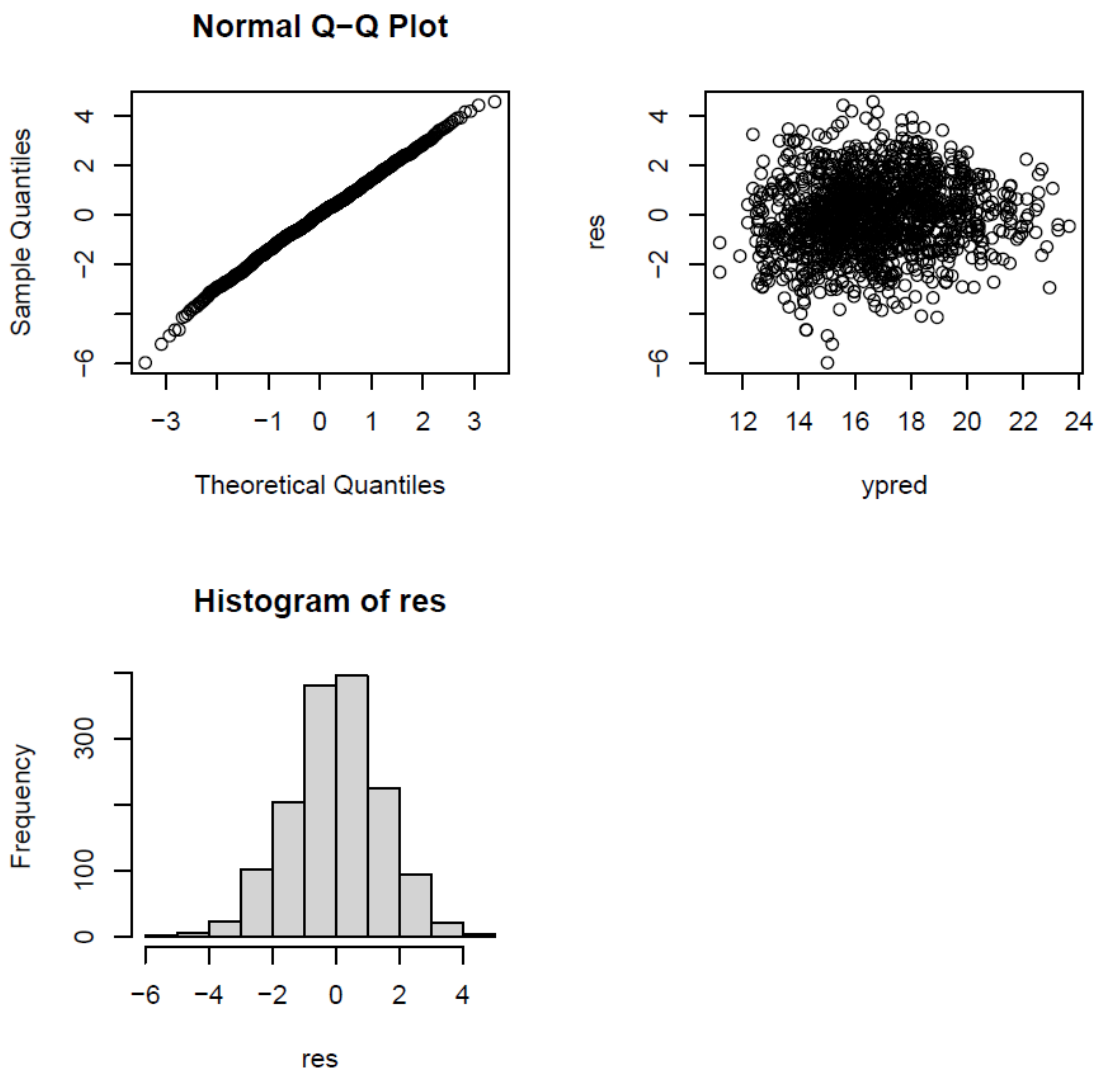

Supplementary Figure 8. **The diagnostic of the linear mixed-effects model (lmer(*Temperature ~ Age*Region* + (1 | *Fish.ID*))).**

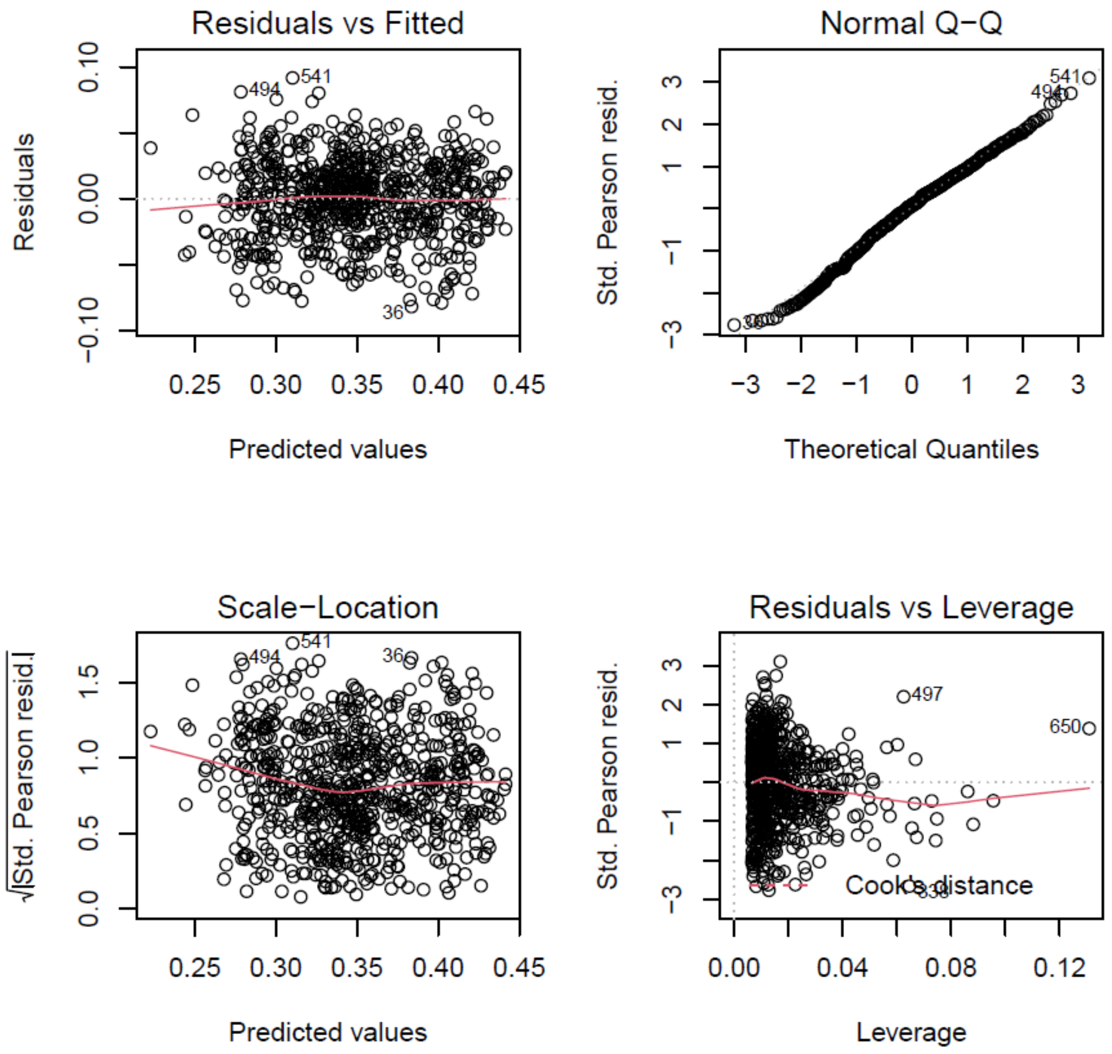

Supplementary Figure 9. The diagnostic of the full model. (M_{oto} ~ glm(*Temperature***Region***Stage*, family=gaussian(link = "identity"))).

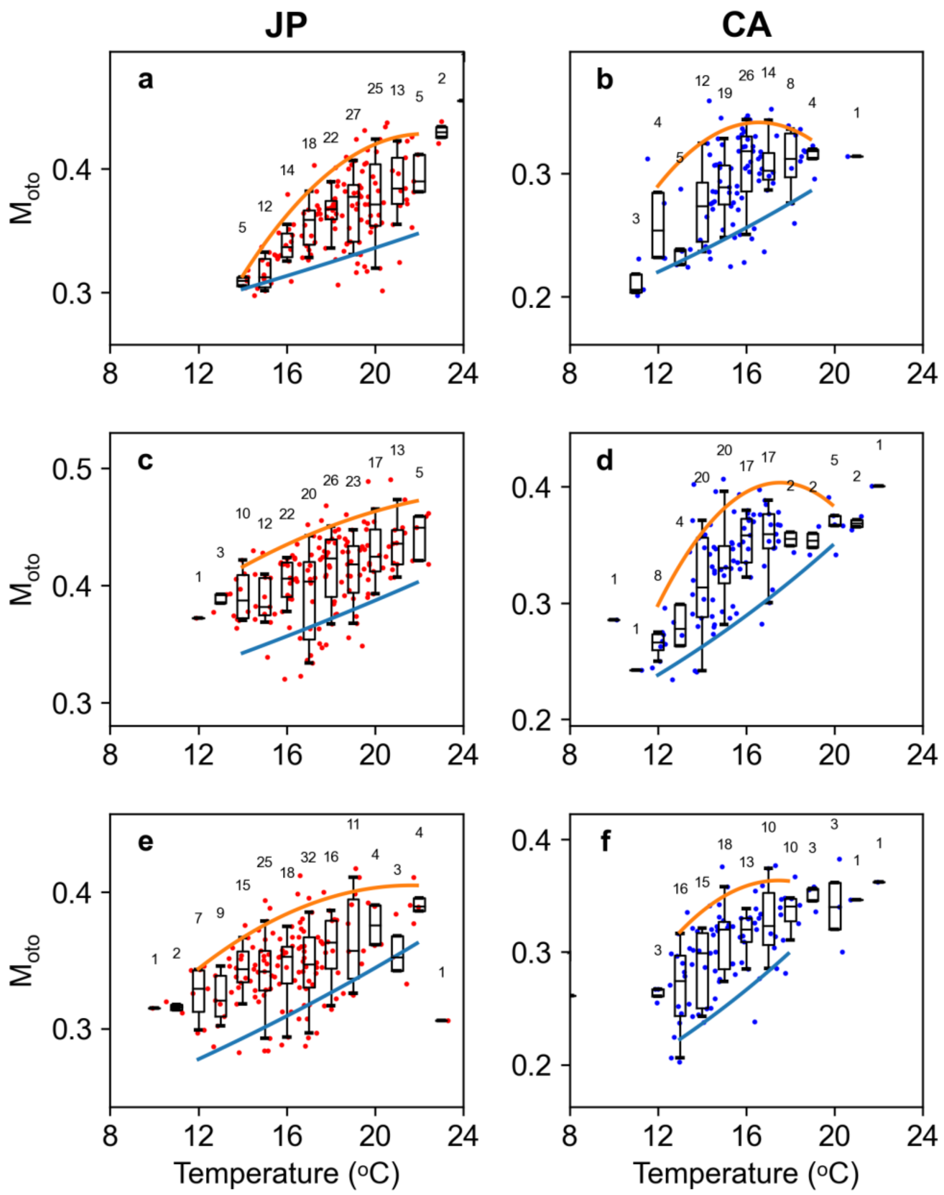

Supplementary Figure 10. Distribution of M_{oto} in each temperature bin. The boxplots of the variation of M_{oto} in each 1 ºC bin for JP sardine during larval (a), early juvenile (c), and late juvenile stages (e) and for CA sardine during larval (b), early juvenile (d), and late juvenile stages (f). Box is 25–75th percentile, the line in the box is median and lower and upper whiskers are 5th and 95th percentiles, respectively. The numbers show the number of individuals for each bin. The orange lines are the polynomial regression for the 95th percentile values of each bin, while the blue lines are the regression for the 5th percentile values calculated by a generalised linear model with gaussian distribution and a log link.

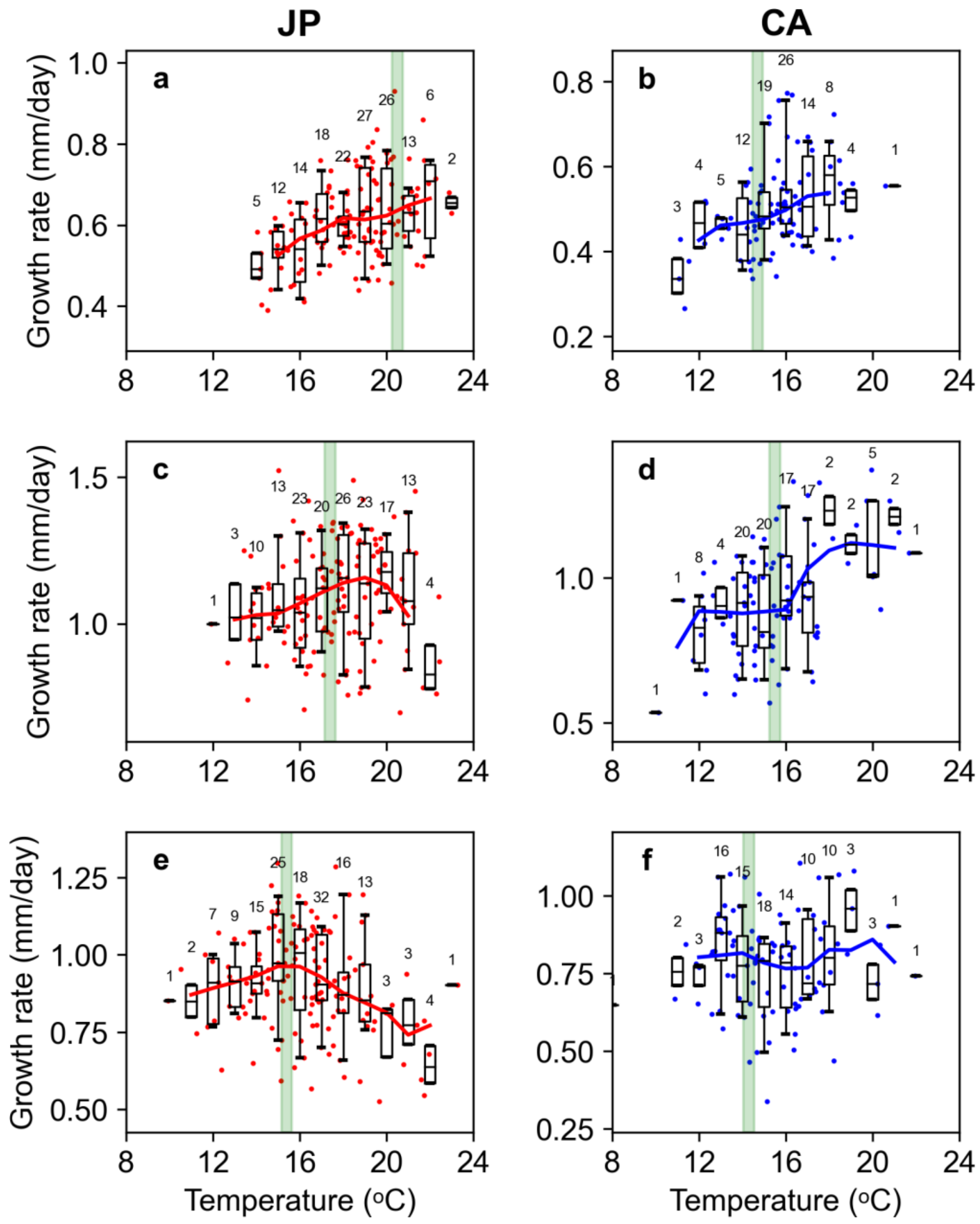

Supplementary Figure 11. Relationship between somatic growth rate and experienced temperature in each stage. The boxplots of the variation of growth rate in each 1 ºC bin for JP sardine during larval (a), early juvenile (c), and late juvenile stages (e) and for CA sardine during larval (b), early juvenile (d), and late juvenile stages (f). Box is 25-75th percentile, the line in the box is median and lower and upper whiskers are 5th and 95th percentiles, respectively. The numbers show the number of individuals for each bin. The red and blue lines are 3-window running means of median of each bin. The green bars are the optimal temperature derived from the analysis of M_{oto}.

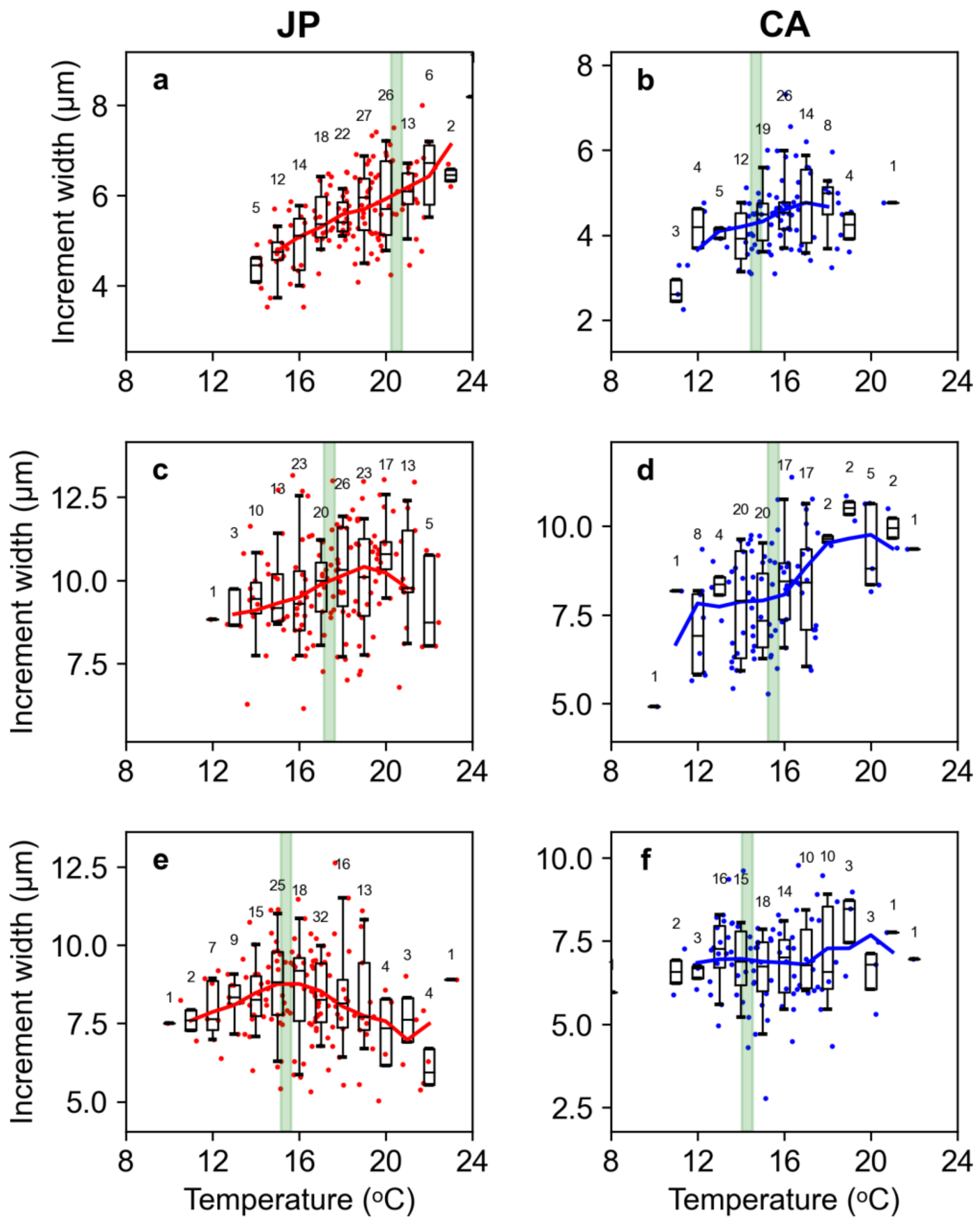

Supplementary Figure 12. Relationship between mean otolith increment width and experienced temperature in each stage. The boxplots of the variation of growth rate in each 1 ºC bin for JP sardine during larval (a), early juvenile (c), and late juvenile stages (e) and for CA sardine during larval (b), early juvenile (d), and late juvenile stages (f). Box is 25-75th percentile, the line in the box is median and lower and upper whiskers are 5th and 95th percentiles, respectively. The numbers show the samples size for each bin. The red and blue lines are 3-window running means of median of each bin. The green bars are the optimal temperature derived from the analysis of M_{oto}.

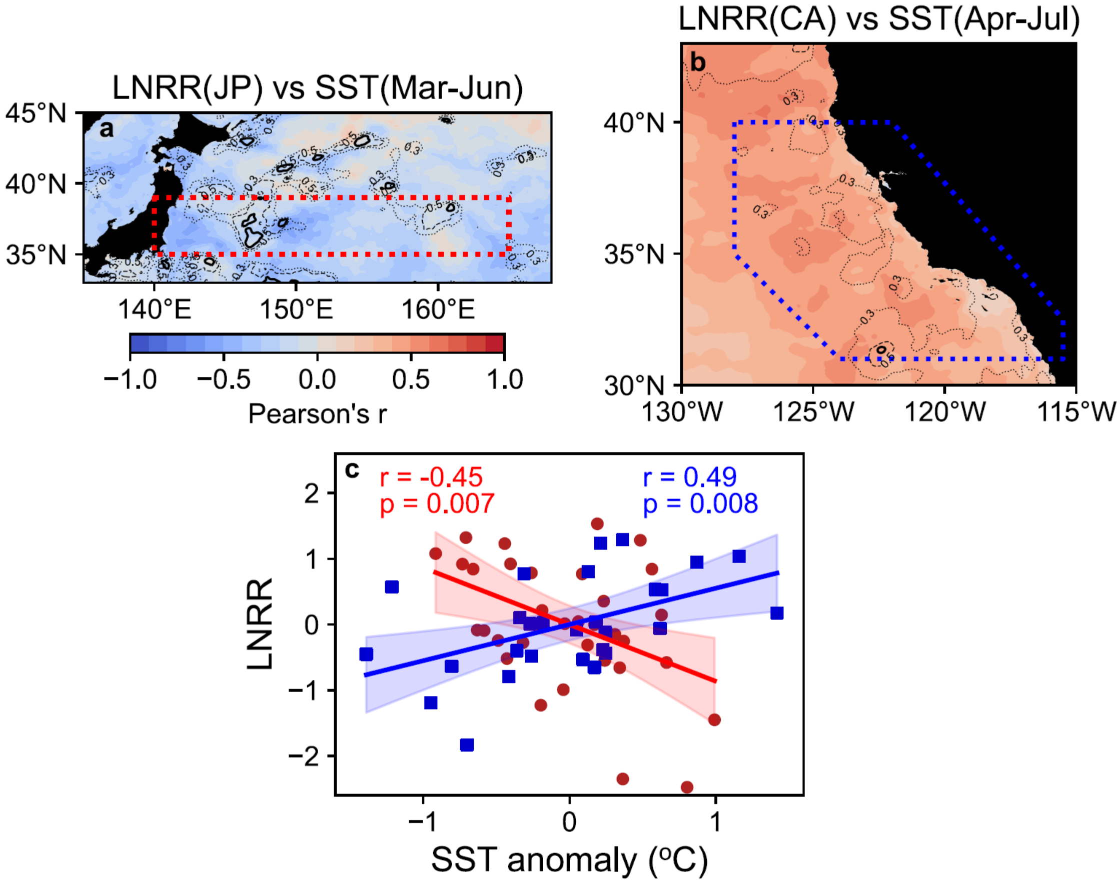

Supplementary Figure 13. Correlations between early life survival and sea surface temperature. (a, b) Pearson's r values calculated between mean sea surface temperature (SST) during March to June and log recruitment residual (LNRR) of JP sardine from 1982 to 2016 (a) and SST during April to July and LNRR of CA sardine from 1982 to 2009 (b) at each grid points. The black contours show areas where Pearson's r values between the SST and year-class mean otolith derived temperature during larval to late juvenile stage were > 0.3 (dotted), > 0.5 (dashed) and higher than statistically significant level ($p < 0.05$ (two-sided but without adjustment for multiple comparison), solid). (c) Relationships between anomalies of SST during March to June in the main habitat area of larvae and juveniles (red- and blue-dotted areas in (a) and (b)) and LNRR of JP (red circles) and CA (blue squares) sardines. Linear regression lines and 95% confidence intervals are shown as solid lines and shades, respectively, together with Pearson's r and p-values (two-sided and without adjustments for multiple comparisons) calculated for sample sizes (number of year-classes) of 35 and 28 for JP and CA sardine, respectively.

Supplementary Table 1. Metadata of JP sardine samples.

Year	Months	Longitude (ºE)	Latitude (ºN)	Station ID	CPUE (inds/30 min)	SL (mean ± 1SD)	N (Microsturucture analysis)	N (Isotope analysis)
2006	9	165.00	45.64	31	31	119.4 ± 5.5	19	6
2006	9	162.06	45.65	36	11	114.0 ± 4.3	9	5
2006	9	162.06	46.30	37	11	114.6 ± 4.2	9	6
2007	9	164.31	45.29	31	11	103.2 ± 5.1	10	6
2007	10	161.95	44.03	37	17	119.0 ± 4.7	16	6
2008	10	161.12	46.98	11	13	113.1 ± 7.8	9	6
2008	10	161.37	47.66	12	10	101.4 ± 7.2	9	6
2008	9	161.12	46.34	9	85	109.0 ± 6.2	19	6
2009	10	165.85	42.92	23	676	119.9 ± 3.6	20	6
2009	9	156.33	44.66	8	504	120.0 ± 6.2	20	6
2010	10	156.36	44.72	18	3995	116.2 ± 4.1	21	6
2010	10	168.15	45.32	19	158	115.3 ± 4.3	14	6
2010	10	163.20	44.53	27	487	115.4 ± 4.9	20	6
2010	10	157.98	43.69	32	2055	117.5 ± 3.4	20	6
2010	9	156.36	44.72	8	673	119.0 ± 7.6	20	6
2014	9	159.80	46.14	11	66.5	129.1 ± 3.8	17	6
2014	9	162.45	46.11	12	95.5	126.6 ± 4.5	20	6
2014	9	162.96	45.56	13	1464	129.2 ± 3.4	20	6
2014	9	163.45	44.97	14	975.5	130.0 ± 3.7	20	6
2014	9	158.97	44.99	9	32	132.9 ± 4.5	16	6
2015	9	166.24	45.46	11	7604	130.0 ± 4.2	20	6
2015	9	156.38	44.68	3	7534	129.2 ± 6.2	13	6
2015	9	158.90	44.96	4	5913	132.8 ± 5.1	20	6
2015	9	159.18	45.33	5	11241	133.7 ± 4.1	20	6
2015	9	162.65	45.82	8	6206	127.6 ± 5.8	10	6
2015	9	162.92	45.53	9	1547	132.3 ± 4	9	7
						(total)	420	156

Supplementary Table 2. Metadata of CA sardine samples.

*The numbers are landing number in California Department of Fish and Wildlife records.

**For samples for which length data were unavailable, standard length was estimated from otolith radius.

Year	Months	Year class	Cruise/ Landing ID* / Port/ Ship	SL (mean ± 1SD)	N (Microsturucture analysis)	N (Isotope analysis)
1987	2	1986	*Nonna Maria II*	145.0**	1	0
1987	5	1986	*Indian*	132.2 ± 5.5**	4	1
1987	1	1986	San Diego	110.9 ± 6.9**	11	6
1991	2	1990	*St. George*	158.8 ± 6.7**	6	3
1992	3	1991	132	144.6 ± 7.5	5	5
1992	6	1991	167	150.8 ± 4.8	4	2
1993	3	1992	194	137.7 ± 7.3	6	5
1993	3	1992	245	152.0 ± 6.1	7	5
1994	4	1993	355	147.5 ± 8.2	6	4
1994	4	1993	427	154.5 ± 2.5	4	4
1994	7	1993	553	157.3 ± 1.5	4	3
1995	3	1994	349	150.0	1	1
1995	3	1994	355	156.8 ± 2.9	5	4
1995	4	1994	504	144.8 ± 5.0	4	4
1995	5	1994	600	158.0	1	1
1996	3	1995	408	146.5 ± 0.7	2	2
1996	4	1995	429	154.0 ± 7.1	2	2
1996	4	1995	503	143.0	1	1
1996	5	1995	531	153.5 ± 4.9	2	1
1997	3	1996	44	133.4 ± 8.8	7	7
1997	4	1996	48	115.0 ± 5.7	2	1
1997	3	1996	55	138.5 ± 6.4	4	2
1997	4	1996	57	122.0	1	1
1998	3	1997	57	110.6 ± 3.2	5	5
1998	5	1997	81	129.8 ± 11.2	4	3
1998	6	1997	88	131.0 ± 4.6	3	2
2005	3	2004	CalCOFI T01	149.0 ± 6.3	10	5

2005	4	2004	CalCOFI T02	145.8 ± 8.3	10	8
2006	1	2005	San Diego	118.3 ± 6.7**	4	4
2007	4	2006	CalCOFI T07	153.3 ± 4.9	4	4
2007	4	2006	CalCOFI T08	136.8 ± 13.5	5	4
				(total)	135	100

Supplementary Table 3. Errors in conversion of otolith δ^{18}O to temperature. Root-mean-square-errors of the formulas converting otolith δ^{18}O to temperature for each month are shown in °C.

Year	Feb	Mar	Apr	May	Jun	Jul	Aug	Sep	Oct
2006	0.7	0.4	0.4	0.5	0.6	0.9	1.4	1.8	2.0
2007	0.6	0.4	0.3	0.4	0.8	0.9	1.2	1.5	1.6
2008	0.9	0.7	0.4	0.5	0.7	1.0	1.4	1.9	1.7
2009	0.7	0.6	0.6	0.5	0.7	0.9	1.2	1.5	1.5
2010	0.6	0.4	0.4	0.3	0.7	10.9	1.4	2.0	1.7
2014	0.7	0.5	0.4	0.5	1.2	1.6	1.5	1.7	1.6
2015	0.5	0.5	0.5	0.6	1.0	1.4	1.6	1.8	1.7

Supplementary Table 4. **Results of pairwise comparison of estimated marginal means of increment widths.** P-values are calculated for two-sided tests and adjusted by Tukey's method for multiple comparisons.

Age	Contrast	Estimate	SE	Cohen's d	df	z ratio	p value
hatch-10	CA - JP	-11.8	1.4	-0.91	Inf	-8.33	**4.31.E-14**
	CA - SA_south-east	-17.8	2.1	-1.37	Inf	-8.29	**4.43.E-14**
	CA - SA_west	-12.9	2.4	-0.99	Inf	-5.46	**2.88.E-07**
	JP - SA_south-east	-6.0	1.9	-0.46	Inf	-3.15	8.80.E-03
	JP - SA_west	-1.1	2.1	-0.08	Inf	-0.49	9.61.E-01
	SA_south-east - SA_west	4.9	2.7	0.38	Inf	1.84	2.55.E-01
**Age**	**Contrast**	**Estimate**	**SE**	**Cohen's d**	**df**	**z ratio**	**p value**
11-20 dph	CA - JP	-16.3	1.4	-1.26	Inf	-11.48	**<2.00.E-16**
	CA - SA_south-east	-24.7	2.1	-1.90	Inf	-11.50	**<2.00.E-16**
	CA - SA_west	-6.9	2.4	-0.53	Inf	-2.93	**1.78.E-02**
	JP - SA_south-east	-8.4	1.9	-0.64	Inf	-4.43	**5.61.E-05**
	JP - SA_west	9.4	2.1	0.72	Inf	4.41	**6.12.E-05**
	SA_south-east - SA_west	17.8	2.7	1.37	Inf	6.65	**1.71.E-10**
**Age**	**Contrast**	**Estimate**	**SE**	**Cohen's d**	**df**	**z ratio**	**p value**
21-30 dph	CA - JP	-22.3	1.4	-1.72	Inf	-15.70	**<2.00.E-16**
	CA - SA_south-east	-25.3	2.1	-1.95	Inf	-11.78	**<2.00.E-16**
	CA - SA_west	-6.6	2.4	-0.51	Inf	-2.79	**2.69.E-02**
	JP - SA_south-east	-3.0	1.9	-0.23	Inf	-1.57	3.97.E-01
	JP - SA_west	15.7	2.1	1.21	Inf	7.38	**9.74.E-13**
	SA_south-east - SA_west	18.7	2.7	1.44	Inf	7.00	**1.54.E-11**
**Age**	**Contrast**	**Estimate**	**SE**	**Cohen's d**	**df**	**z ratio**	**p value**
31-40 dph	CA - JP	-25.6	1.4	-1.97	Inf	-18.02	**<2.00.E-16**
	CA - SA_south-east	-28.8	2.1	-2.21	Inf	-13.40	**<2.00.E-16**
	CA - SA_west	-3.9	2.4	-0.30	Inf	-1.66	3.47.E-01
	JP - SA_south-east	-3.1	1.9	-0.24	Inf	-1.66	3.44.E-01
	JP - SA_west	21.7	2.1	1.67	Inf	10.19	**3.59.E-14**
	SA_south-east - SA_west	24.9	2.7	1.91	Inf	9.31	**4.13.E-14**
**Age**	**Contrast**	**Estimate**	**SE**	**Cohen's d**	**df**	**z ratio**	**p value**
41-50 dph	CA - JP	-29.6	1.4	-2.28	Inf	-20.83	**<2.00.E-16**
	CA - SA_south-east	-29.5	2.1	-2.27	Inf	-13.75	**<2.00.E-16**

Age	Contrast	Estimate	SE	Cohen's d	df	z ratio	p value
	CA - SA_west	-1.5	2.4	-0.12	Inf	-0.64	9.18.E-01
	JP - SA_south-east	0.1	1.9	0.01	Inf	0.04	1.00.E+00
	JP - SA_west	28.1	2.1	2.16	Inf	13.19	**0.00.E+00**
	SA_south-east - SA_west	28.0	2.7	2.15	Inf	10.49	**3.51.E-14**
**Age**	**Contrast**	**Estimate**	**SE**	**Cohen's d**	**df**	**z ratio**	**p value**
51-60 dph	CA - JP	-30.2	1.4	-2.33	Inf	-21.27	**<2.00.E-16**
	CA - SA_south-east	-24.1	2.1	-1.85	Inf	-11.22	**8.88.E-15**
	CA - SA_west	4.9	2.4	0.37	Inf	2.05	1.70.E-01
	JP - SA_south-east	6.1	1.9	0.47	Inf	3.25	**6.36.E-03**
	JP - SA_west	35.1	2.1	2.70	Inf	16.35	**<2.00.E-16**
	SA_south-east - SA_west	29.0	2.7	2.23	Inf	10.79	**3.80.E-14**
**Age**	**Contrast**	**Estimate**	**SE**	**Cohen's d**	**df**	**z ratio**	**p value**
61-70 dph	CA - JP	-25.3	1.4	-1.95	Inf	-17.81	**<2.00.E-16**
	CA - SA_south-east	-13.2	2.2	-1.02	Inf	-6.13	**5.20.E-09**
	CA - SA_west	9.1	2.4	0.70	Inf	3.83	**7.30.E-04**
	JP - SA_south-east	12.1	1.9	0.93	Inf	6.36	**1.22.E-09**
	JP - SA_west	34.4	2.1	2.65	Inf	16.03	**<2.00.E-16**
	SA_south-east - SA_west	22.3	2.7	1.72	Inf	8.30	**4.45.E-14**
**Age**	**Contrast**	**Estimate**	**SE**	**Cohen's d**	**df**	**z ratio**	**p value**
71-80 dph	CA - JP	-17.3	1.4	-1.33	Inf	-12.18	**<2.00.E-16**
	CA - SA_south-east	-3.4	2.2	-0.26	Inf	-1.55	4.08.E-01
	CA - SA_west	10.6	2.4	0.81	Inf	4.45	**5.09.E-05**
	JP - SA_south-east	14.0	1.9	1.07	Inf	7.30	**1.77.E-12**
	JP - SA_west	27.9	2.1	2.15	Inf	12.99	**<2.00.E-16**
	SA_south-east - SA_west	13.9	2.7	1.07	Inf	5.16	**1.46.E-06**
**Age**	**Contrast**	**Estimate**	**SE**	**Effect size**	**df**	**z ratio**	**p value**
81-90 dph	CA - JP	-9.1	1.4	-0.70	Inf	-6.37	**1.16.E-09**
	CA - SA_south-east	1.6	2.2	0.12	Inf	0.73	8.84.E-01
	CA - SA_west	7.2	2.5	0.55	Inf	2.91	**1.91.E-02**
	JP - SA_south-east	10.7	1.9	0.82	Inf	5.53	**1.87.E-07**
	JP - SA_west	16.2	2.2	1.25	Inf	7.23	**2.83.E-12**
	SA_south-east - SA_west	5.6	2.8	0.43	Inf	2.00	1.88.E-01
**Age**	**Contrast**	**Estimate**	**SE**	**Cohen's d**	**df**	**z ratio**	**p value**
91-100 dph	CA - JP	-2.3	1.4	-0.18	Inf	-1.64	3.58.E-01
	CA - SA_south-east	5.6	2.2	0.43	Inf	2.56	5.09.E-02

CA - SA_west	4.6	2.6	0.36	Inf	1.76	2.94.E-01
JP - SA_south-east	8.0	1.9	0.61	Inf	4.09	**2.56.E-04**
JP - SA_west	7.0	2.4	0.53	Inf	2.87	**2.17.E-02**
SA_south-east - SA_west	-1.0	3.0	-0.08	Inf	-0.34	9.86.E-01

Supplementary Table 5. Results of pairwise comparison of estimated marginal means of M_{oto}. P-values are calculated for two-sided tests and adjusted by Tukey's method for multiple comparisons.

Age	Contrast	estimate	SE	Cohens'd	df	t ratio	p value
0-30	CA - JP	-0.0742	0.0052	-2.37	860	-14.22	**2.17.E-41**
31-60	CA - JP	-0.1018	0.0049	-3.24	708	-20.96	**2.98.E-76**
61-90	CA - JP	-0.0437	0.0048	-1.39	699	-9.06	**1.32.E-18**
91-120	CA - JP	-0.0172	0.0049	-0.55	721	-3.52	**4.56.E-04**

Supplementary Table 6. Results of pairwise comparison of estimated marginal means of the experienced temperature. P-values are calculated for two-sided tests and adjusted by Tukey's method for multiple comparisons.

Age	Contrast	estimate	SE	Cohen's d	df	t ratio	p value
0-30	CA - JP	-2.81	0.31	-1.77	582	-8.95	**4.91.E-18**
31-60	CA - JP	-2.73	0.30	-1.72	492	-9.13	**1.70.E-18**
61-90	CA - JP	-1.50	0.30	-0.94	485	-5.04	**6.63.E-07**
91-120	CA - JP	-0.10	0.30	-0.06	501	-0.33	7.44.E-01

Supplementary Table 7. AIC based selection of generalised linear models to explain M_{oto} variation.

Models	AIC
M_{oto} ~ glm(*Temperature***Region*, family=gasussian(link = "log"))	-2737.195
M_{oto} ~ glm(*Temperature***Region*, family=gasussian(link = "identity"))	-2740.131
M_{oto} ~ glm(*Temperature* + *Region*, family=gasussian(link = "identity"))	-2736.767
**M_{oto} ~ glm(*Temperature***Region***Stage*, family=gasussian(link = "identity"))**	**-3059.121**
M_{oto} ~ glm(*Temperature***Region* + *Stage*, family=gasussian(link = "identity"))	-3034.695

Supplementary Table 8. Summary table of coefficients of the full model. (M_{oto} ~ glm(Temperature*Region*Stage, family=gaussian(link = "identity"))). P-values are calculated for two-sided tests without adjustments for multiple comparisons.

Coefficients

**Factors**	**Estimate**	**Std. Error**	**t value**	**P value**
(Intercept)	0.1264	0.0262	4.824	**1.72E-06**
Temperature	0.01061	0.001669	6.356	**3.66E-10**
Region (JP)	0.03302	0.03395	0.972	3.31.E-01
Stage (early juvenile)	0.01986	0.03346	0.594	5.53.E-01
Stage (late juvenile)	0.01509	0.0334	0.452	6.52.E-01
Temperature:Region (JP)	0.0004228	0.002037	0.208	8.36.E-01
Temperature:Stage (early juvenile)	0.00144	0.002134	0.675	5.00.E-01
Temperature:Stage (late juvenile)	4.625E-05	0.002133	0.022	9.83.E-01
Region (JP):Stage (early juvenile)	0.1098	0.0443	2.479	**1.34.E-02**
Region (JP):Stage (late juvenile)	0.09093	0.04305	2.112	**3.50.E-02**
Temperature:Region (JP):Stage (early juvenile)	-0.005673	0.002665	-2.128	**3.36.E-02**
Temperature:Region (JP):Stage (late juvenile)	-0.006052	0.002633	-2.299	**2.18.E-02**

Supplementary Table 9. **Correlations between fish size and temperature.** Pearson's r and raw p-values (two-sided) calculated for standard length and mean temperature at each age and corrected p-values using the Benjamini-Hochberg procedure with $\alpha = 0.05$. Note that the raw p-value for JP sardine at 75 dph (*) is that of the coefficients of the quadratic term in the linear model (Supplementary Table 10).

**Region**	**JP**	**JP**	**JP**	**CA**	**CA**	**CA**
**Age (dph)**	**45**	**75**	**105**	**60**	**90**	**120**
**Pearson's r**	0.46	0.08	-0.58	0.42	0.47	0.43
**p-value (raw)**	0.018	0.038*	0.002	0.046	0.023	0.040
**p-value (corrected)**	0.045	0.046	0.011	0.046	0.045	0.046
**rejection**	TRUE	TRUE	TRUE	TRUE	TRUE	TRUE

Supplementary Table 10. Summary table of coefficients of the best linear model (*Standard Length ~ Temperature*^{2} + *Temperature*) for JP sardine at 75 dph (early juvenile). P-values are calculated for two-sided tests without adjustments for multiple comparisons.

**Coefficients**				
**Factors**	**Estimated**	**Std. Error**	**t-value**	**p-value**
**Intercept**	-150.285	96.397	-1.56	0.133
**Temperature**	23.596	10.631	2.22	0.037
**(Temperature)**2**	-0.642	0.292	-2.20	0.038
