# Supplementary material for: Contrasting life-history responses to climate variability in eastern and western North Pacific sardine populations
Source: Nat Commun. 2022 Oct 16;13:5298. doi: 10.1038/s41467-022-33019-z (PMC9573866; doi:10.1038/s41467-022-33019-z)
Supplement: Supplementary file 3 — Reporting Summary [file 41467_2022_33019_MOESM3_ESM.pdf]

nature research

Corresponding author(s): Tatsuya Sakamoto

Last updated by author(s): Aug 16, 2022

Reporting Summary

Nature Research wishes to improve the reproducibility of the work that we publish. This form provides structure for consistency and transparency in reporting. For further information on Nature Research policies, see our Editorial Policies and the Editorial Policy Checklist.

Statistics

For all statistical analyses, confirm that the following items are present in the figure legend, table legend, main text, or Methods section.

n/a	Confirmed	
☐	☒	The exact sample size (n) for each experimental group/condition, given as a discrete number and unit of measurement
☐	☒	A statement on whether measurements were taken from distinct samples or whether the same sample was measured repeatedly
☐	☒	The statistical test(s) used AND whether they are one- or two-sided<br>*Only common tests should be described solely by name; describe more complex techniques in the Methods section.*
☐	☒	A description of all covariates tested
☐	☒	A description of any assumptions or corrections, such as tests of normality and adjustment for multiple comparisons
☐	☒	A full description of the statistical parameters including central tendency (e.g. means) or other basic estimates (e.g. regression coefficient) AND variation (e.g. standard deviation) or associated estimates of uncertainty (e.g. confidence intervals)
☐	☒	For null hypothesis testing, the test statistic (e.g. F, t, r) with confidence intervals, effect sizes, degrees of freedom and P value noted<br>*Give P values as exact values whenever suitable.*
☒	☐	For Bayesian analysis, information on the choice of priors and Markov chain Monte Carlo settings
☒	☐	For hierarchical and complex designs, identification of the appropriate level for tests and full reporting of outcomes
☐	☒	Estimates of effect sizes (e.g. Cohen's d, Pearson's r), indicating how they were calculated

Our web collection on statistics for biologists contains articles on many of the points above.

Software and code

Policy information about availability of computer code

Data collection	Software specific to mass spectrometers (IonVantage, Isodat) and custom Excel sheets were used for calculating isotope ratios.
Data analysis	Custom Python 3.9.7, R 4.1.3, MATLAB R2017a and Generic Mapping Tools 4.5.11 codes were developed for analyses and data visualization. The custom codes is deposited in Zenodo https://doi.org/10.5281/zenodo.6983520.

For manuscripts utilizing custom algorithms or software that are central to the research but not yet described in published literature, software must be made available to editors and reviewers. We strongly encourage code deposition in a community repository (e.g. GitHub). See the Nature Research guidelines for submitting code & software for further information.

Data

Policy information about availability of data

All manuscripts must include a data availability statement. This statement should provide the following information, where applicable:

- Accession codes, unique identifiers, or web links for publicly available datasets
- A list of figures that have associated raw data
- A description of any restrictions on data availability

The otolith and seawater isotope ratio and otolith microstructure data generated in this study have been deposited in the Dryad under accession code https://doi.org/10.5061/dryad.5mkkwh78j. PDO index data can be downloaded from https://www.ncei.noaa.gov/access/monitoring/pdo/. CUTI data can be downloaded from https://mjacox.com/upwelling-indices/. The data of stable carbon isotope ratio of DIC can be downloaded from https://www.ncei.noaa.gov/products/world-ocean-database. CalCOFI hydrographic data can be downloaded from https://calcofi.org/data/.The Argo float data can be downloaded from http://www.jamstec.go.jp/e/database/. The sea surface temperature products can be downloaded from https://resources.marine.copernicus.eu/product-detail/SST_GLO_SST_L4_REP_OBSERVATIONS_010_011/INFORMATION.

Field-specific reporting

Please select the one below that is the best fit for your research. If you are not sure, read the appropriate sections before making your selection.

☐ Life sciences ☐ Behavioural & social sciences ☒ Ecological, evolutionary & environmental sciences

For a reference copy of the document with all sections, see nature.com/documents/nr-reporting-summary-flat.pdf

Ecological, evolutionary & environmental sciences study design

All studies must disclose on these points even when the disclosure is negative.

Study description	We analysed microstructure, carbon and oxygen stable isotope ratios of otoliths of sardines in the western and eastern North Pacific. The measurements provided the somatic growth, metabolic rate, and temperature histories of individuals during their early life stages. The histories and relationships between them were compared among the populations to understand why the populations exhibit opposite responses to decadal scale anomalies in ocean temperature.
Research sample	We focused on the pacific subpopulation of Japanese sardine Sardinops sagax melanostictus (Family, Clupeidae) and the Northern subpopulation of Pacific sardine Sardinops sagax sagax (Family, Clupeidae). These are the subpopulations that are most responsible for fluctuations of sardine biomass in the western and eastern North Pacific, and of great commercial and ecological importance. Japanese sardine samples were age-0 captured during cruise surveys in the subarctic Western North Pacific, which represent the recruits of the the Pacific subpopulation of Japanese sardine. The Pacific sardine samples were age-1 captured in cruise surveys and in the pelagic fishery of the Southern California Bight, which represent the recruits of the the Northern subpopulation of Pacific sardine. Sex of the fish were mostly not determined.
Sampling strategy	Because this study used archived otoliths that had been collected before the research started, the sample size was limited by the number of samples archived. Within this limitation, a hundred or more individuals in total from more than twenty sampling batches were used for isotope analyses for each population, which allowed regression analyses in individual and sampling batch levels. Otolith isotope analyses were conducted in 15 or 30 days temporal resolution, which was sufficiently high to resolve ontogenetic shifts of thermal and metabolic traits of individuals.
Data collection	Otoliths were embedded into resin and then ground to expose the core. After observation of microstructure, they were micromilled to obtain powders for isotope analysis. Isotope data were acquired during 2016 to 2018 at the Atmosphere and Ocean Research Institute of the University of Tokyo using DELTA V + GAS Bench system, or at the National Institute of Technology of Ibaraki College using MICAL 3c by Tatsuya Sakamoto, Toyoho Ishimura, Kotaro Shirai, and Tomihiko Higuchi.
Timing and spatial scale	Otoliths were collected from age-0 recruited Japanese sardine sampled in the Oyashio region (155-170E, 42-48N) during 2006–2010, 2014, and 2015 and from recruited age-1 Pacific sardine captured in the coastal waters of the Southern California Bight (115-125W, 30-35N) during 1987, 1991–1998, and 2005–2007. Isotope data were acquired during 2016 to 2018. The regions are the main recruitment area of each population, and the sampling years for both populations included the periods of biomass increases, thereby allowing discussion about the factors that drives the increase of the populations.
Data exclusions	Isotope data were excluded when the height of sample peak was below the level of which precision is guaranteed during mass spectrometry. In the estimation of optimal temperature based on the otolith-derived metabolic proxy Moto, outliers detected using the boxplot function in R4.1.3 were excluded. In the comparison between fish size and mean experienced temperature, individuals that had missing data at any age range were excluded.
Reproducibility	As data and codes supporting this study are published in Dryad without any restrictions for further use, the results are fully reproducible.
Randomization	Individuals for analyses were randomly chosen from fishes of the target age (0 for Japanese sardine and 1 for Pacific sardine) in each sampling batch.
Blinding	Blinding was not relevant to this study because the isotope analyses are unlikely to be affected by experimenter bias. Instead, the measurement orders of samples were explicitly shuffled to avoid systematic bias due to machine drifts.

Did the study involve field work? ☐ Yes ☒ No

Reporting for specific materials, systems and methods

We require information from authors about some types of materials, experimental systems and methods used in many studies. Here, indicate whether each material, system or method listed is relevant to your study. If you are not sure if a list item applies to your research, read the appropriate section before selecting a response.

Materials & experimental systems

n/a	Involved in the study	
x		Antibodies
x		Eukaryotic cell lines
x		Palaeontology and archaeology
	x	Animals and other organisms
x		Human research participants
x		Clinical data
x		Dual use research of concern

Methods

n/a	Involved in the study	
x		ChIP-seq
x		Flow cytometry
x		MRI-based neuroimaging

Animals and other organisms

Policy information about studies involving animals; ARRIVE guidelines recommended for reporting animal research

Laboratory animals	This study did not involve laboratory animals.
Wild animals	This study did not involve wild animals.
Field-collected samples	Fish collected in the field were frozen on board or at port and thawed in the laboratory for dissection. Extracted otoliths were dried and kept at room temperature.
Ethics oversight	All procedures accorded to administrative provision of animal welfare of the Fisheries Research Education Agency Japan.

Note that full information on the approval of the study protocol must also be provided in the manuscript.
